# Unblinding at disease progression in double-blinded randomized controlled cancer drug clinical trials: A controversy requires more attention

**DOI:** 10.3389/fmed.2022.1082445

**Published:** 2022-12-13

**Authors:** Wu Dawei, Miao Shuangman, Huang Huiyao, Cui Dandan, Tang Yu, Li Ning

**Affiliations:** Department of Clinical Trials Center, National Cancer Center/National Clinical Research Center for Cancer/Cancer Hospital, Chinese Academy of Medical Sciences and Peking Union Medical College, Beijing, China

**Keywords:** unblinding, disease progression, ethics, double-blinded randomized controlled trials, clinical trials design

## Abstract

Unblinding at disease progression in double-blinded randomized controlled cancer drug clinical trials is ethical to the patient by ensuring optimal subsequent treatment, but the effect of study treatment on overall survival may be confounded. The views of science and ethics in this issue are controversial and the unblinding procedures should be well-designed. In real world settings, a lack of use of this unblinding process in protocol was observed in the analysis of 134 double-blind randomized controlled anticancer drug clinical trials conducted in China from 2018 to 2021. Unblinding at disease progression was allowed in only 26 (18.2%) trials. Among them, Only 9 (34.6%) trials involved patient-level unblinding. None of the 134 included trials accounted for the risk of blind-maintenance after disease progression. Based on the analysis and case studies, we believe that unblinding at disease progression should be stated in the protocol when the treatment assignment directly affected the choice of subsequent regimen, in which the drug category, control group design, standard of care of further-line therapy and primary endpoint together play a role. When unblinding at disease progression is adopted, the sensitivity analytics are recommended to understand the true effect of study drug on overall survival. The notification of treatment allocation after unblinding and the informed consent also require attention. A decision-making framework is established to help understand this controversy, which should be carefully discussed by the investigator and the sponsor.

## Introduction

The double-blinded randomized controlled trial (RCT) is a golden standard in the clinical assessment of novel drugs. Blinding investigators and patients decreases the likelihood of biased observations of the effectiveness outcomes and the patient drop out (1). Withholding the participants' treatment assignment may adversely affect their medical care in case of emergency (2) or after the completion of study (3, 4), thus raises ethical concerns. The unblinding procedure is well-established in trial protocol in response to these circumstances. However, in double-blinded randomized controlled cancer drug clinical trials, maintaining the blind at disease progression may also have a negative impact on the subjects' subsequent treatment, namely, preventing or delaying them from receiving approved therapy or entering into other clinical trials (1). Whether and how to set the unblinding procedure in this situation require further discussion.

From a policy perspective, this issue seems ignored by current regulations and guidelines, which are mainly aimed at unblinding under emergency or at the end of study (2–4). Only Food and Drug Administration (FDA) of the United States has issued relative guidance, which from the ethical view, recommended unblinding the patient and the investigator at disease progression or recurrence to ensure optimal subsequent management. The guidance also emphasized the informed consent of relevant risk if the sponsor intended to maintain patient-level blinding at disease progression (1). As only placebo-controlled studies are within the scope of guidance, it is unclear whether this recommendation should be adopted in other double-blinded cancer drug RCTs.

To make matters worse, it is reported that pharmaceutical industry trials most often maintain blinding until the completion of the entire study (5). For cancer clinical trials, unblinding process at disease progression or crossover design is avoided when OS is part of the primary endpoint. The sponsors, from the scientific view, claim that the reliability of OS endpoint is negatively impacted by the open-label stage beyond disease progression, and the effect of study treatment is potentially confounded by switch-over from control to study treatment or subsequent therapies (6, 7). Therefore, the rules or procedures should be established to balance the paradoxical views of science and ethics at this question.

## Cross-sectional study

In order to clarify whether the blinding is maintained within disease progression at trial level, and how the procedures are designed in real world settings, a total of 134 double-blinded randomized controlled cancer drug clinical trials launched in China from 2018 to 2021 were analyzed. Based on the protocols, except for 2 (1.5%) trials without any unblinding process, unblinding at disease progression was allowed and stated in 26 (18.2%) trials. Most trials (106, 74.1%) only had an unblinding process under emergency or after the final analysis of study.

The 26 trials with unblinding process at disease progression were all testing novel drugs, which accounted for 20.1% of all the trials for this drug category. For sponsor type, a higher proportion of trials sponsored by international cooperations (13/48, 27.1%) included such procedures, compared with that of domestic cooperations (13/86, 15.1%). Placebo-controlled trials (24/112, 21.4%), without OS as the primary endpoint (20/99, 20.2%) were more likely to adopt the procedures than trials within active comparators (2/21, 9.5%), and OS as the primary endpoint (6/35, 17.1%). The χ^2^ test was used for subgroup comparisons, but no statistically significance was observed (Table 1).

**Table 1:** The distribution of unblinding procedures by drug category, sponsor type, control group design and primary endpoint of the 134 trials.

**Unblinding at disease progression stated in protocol**	**Yes (*n* = 26)**	**No (*n* = 108)**	***P*** **-value**
**Drug category**			
Novel drugs (*n* = 124)	26 (26/124, 20.1%)	98 (98/124, 79.0%)	*P* = 0.11
Generics / biosimilars (*n* = 10)	0	10 (10/10, 100%)	
**Sponsor type**			
Domestic (*n* = 86)	13 (13/86, 15.1%)	73 (73/86, 84.9%)	*P* = 0.09
International (*n* = 48)	13 (13/48, 27.1%)	35 (35/48, 72.9%)	
**Control group design**			
Placebo / add-on design (*n* = 113)	24 (24/112, 21.4%)	89 (89/112, 79.5%)	*P* = 0.21
Active comparators (*n* = 21)	2 (2/21, 9.5%)	19 (19/21, 90.5%)	
**Primary endpoint**			
OS included (*n* = 35)	6 (6/35, 17.1%)	29 (29/35, 82.9%)	*P* = 0.69
OS not included (*n* = 99)	20 (20/99, 20.2%)	79 (79/99, 79.8%)	

OS, Overall survival.

Among the 26 protocols with unblinding procedures at disease progression, the patients would receive standard of care (SOC) after unblinding in 16 (61.6%) trials. The patients in control group would crossover to the study treatment after unblinding in the other 10 (38.5%) trials. As for the disclosure of treatment assignment after unblinding, there were 13 (50.0%) trials at the investigator level, 7 (26.9%) at both the investigator and the patient level, 4 (15.4%) at both the investigator and the sponsor level, 2 (7.7%) unblinded all the three parties. Only 9 (34.6%) trials in accumulation involved patient-level unblinding. Looking at the informed consent documents, unfortunately, none of the included clinical trials accounted for the risk of blind-maintenance after disease progression.

## Discussion

The data demonstrated that unblinding at disease progression was not received enough attention in cancer clinical trials, though it is beneficial to patients and supported by the FDA guidance. In order to better reshape the unblinding rules, the influence of blinding on subsequent treatment is the most important factor. The analysis has given us typical cases for how blind-maintenance at disease progression affect the medical care, in which the drug category, control group design and SOC of further-line therapy together play a role (Supplementary Figure 1). In Case A: the first-line study treatment of non-small-cell lung cancer (NSCLC), when the study drug is a biosimilar, patients receive “similar” study treatment that doesn't affect the choice of the second-line therapy. Maintaining blinding at disease progression is acceptable. In Case B: the first-line study treatment of esophageal squamous cell carcinoma (ESCC), when the trial is placebo-controlled, patients receive totally “different” study treatment. Also, the SOC of the second-line shares the same target of the study drug. The treatment assignment directly affected the choice of subsequent regimen, thus unblinding should be considered.

The OS will obviously be impacted by the unblinding progress in trials like Case B. Due to patient welfare, early unblinding and crossover treatment should still be considered (8). To reduce the effect on the trial data interpretation, sometimes the primary endpoint may be carefully re-designed and a discussion with the regulatory authority is necessary. When the primary endpoint involves OS and the study treatment does not directly affect the subsequent care, unblinding at disease progression at trial level is not mandatory, but unblinding individual patient may still be considered. The sensitivity analytics with modeling of crossover impact are recommended in the above cases whenever unblinding occurs in a group of patients or an individual, to understand the true benefit of study drug and meet the requirements of regulatory authorities (9).

Simply replacing the rules of unblinding at disease progression by emergency unblinding or unblinding upon the completion of study is not recommended. Firstly, the “disease progression” is not necessarily an “emergency.” Secondly, “completion of study treatment” is far from “completion of study” for cancer patients (5), especially when overall survival (OS) is collected as a key endpoint. Finally, without a detailed standard procedure, the degree of operating freedom is too large to fully protect the welfare of patients.

The notification of treatment allocation after unblinding and the informed consent cannot be ignored. The treatment assignment must be disclosed at the patient level, especially when the subsequent care is provided by other physicians rather than the investigators (10). The risk of blinding (e.g., delaying the subsequent medical care) and the conditions of unblinding should be addressed in the written informed consent and reviewed by the ethics committee. If the patient-level blindness is maintained at disease progression, the additional risk should also be reflected in the informed consent (1).

**Figure 1:**
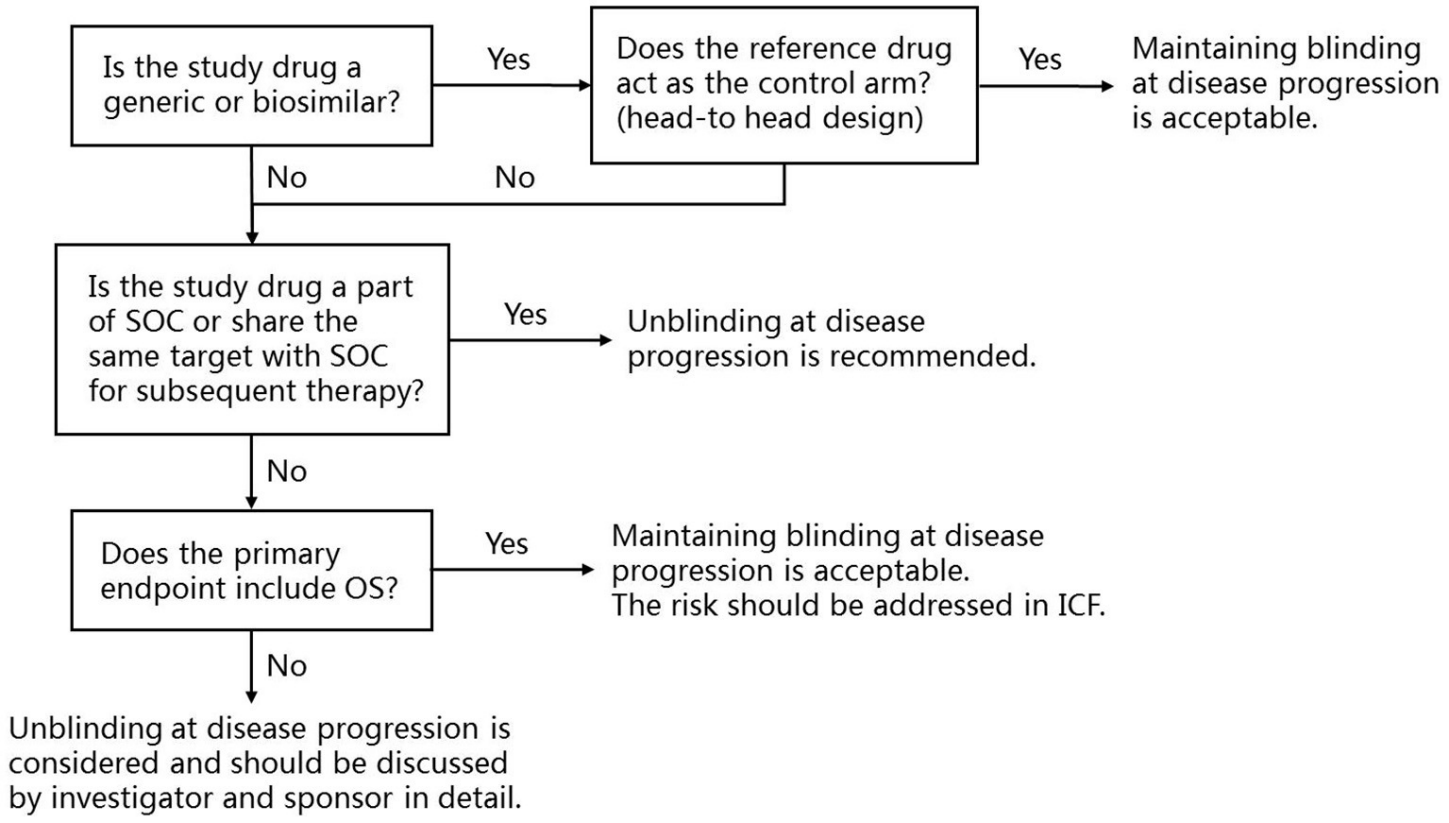
The framework for the application of unblinding at disease progression at trial level and recommendations. SOC, Standard of care; OS, Overall survival; ICF, Informed consent form.

Based on the above considerations, a framework for protocol design is established to help sponsors, investigators and ethics committees to understand the controversy (Figure 1). When the study design is a head-to-head comparison of a generic or biosimilar and its reference drug, maintaining blinding at disease progression is acceptable. For novel cancer drug clinical trials, when the study drug is part of, or share the same target of subsequent SOC, unblinding at disease progression is recommended. When the study treatment does not directly affect the subsequent therapy, and the primary endpoint includes OS, maintaining blinding at disease progression is acceptable, but the risk should be addressed in the written informed consent.

In summary, the application of unblinding procedures at disease progression in double-blinded cancer drug RCTs has not been paid enough attention and should be carefully reshaped by the investigator and the sponsor under patient-centric considerations. To fully establish the decision-making mechanism in the future, additional studies are needed, such as surveys on the awareness of different stakeholders to this issue, implementation of the unblinding procedures at patient level in real world, and cohort studies exploring the effect of blinding to the prognosis of trial participants.

## Data availability statement

The original contributions presented in the study are included in the article/Supplementary material, further inquiries can be directed to the corresponding authors.

## Author contributions

The initial conceptual framework for the viewpoint was developed by WD together with TY and LN, and WD, MS, HH, CD, TY, and LN collaborated in refining it. The first draft of the article was written by WD and MS. Literature search and data collection were done by MS. The figures in this article were drawn by WD. Data analysis and data interpretation were accessed by CD and HH separately. All authors contributed to the article and approved the submitted version.
