# Supplementary material for: Unblinding at disease progression in double-blinded randomized controlled cancer drug clinical trials: A controversy requires more attention
Source: Front Med (Lausanne). 2022 Dec 13;9:1082445. doi: 10.3389/fmed.2022.1082445 (PMC9792660; doi:10.3389/fmed.2022.1082445)
Supplement: Supplementary file 1 [file Table_1.DOCX]

**Supplemental Material**


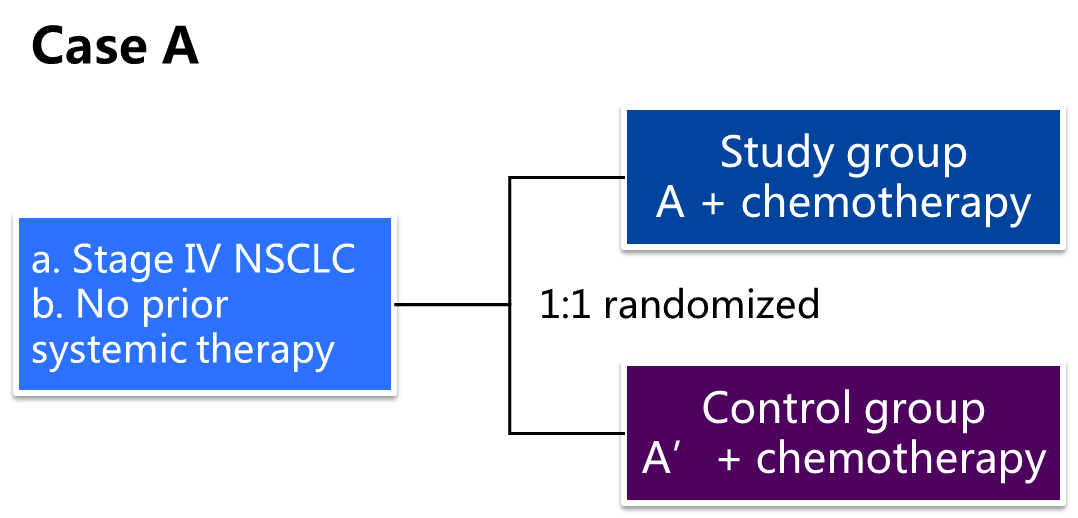


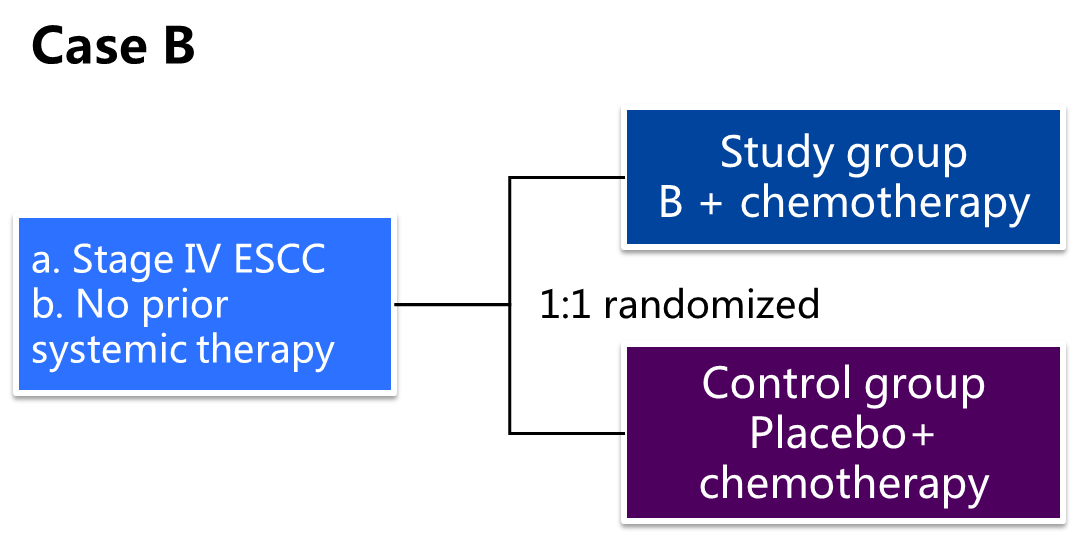


**Figure S1:** Case study for when unblinding at disease progression should be recommended

**Case A:** The trial targets first-line therapy of NSCLC. Study drug A is the biosimilar of reference drug A’. Maintaining blinding at disease progression in this trial is acceptable. **Case B:** The trial targets first-line therapy of ESCC. Study drug B shares the same target of approved drug B’, and drug B’ is the standard of care for the second-line treatment of ESCC. Unblinding at disease progression is recommended in this trial.

NSCLC: Non small-cell lung cancer; ESCC: Esophageal squamous cell carcinoma
